# Microbiota and Guillain-Barré syndrome: role of microbial metabolites, biomarkers, and emerging therapeutic strategies

**DOI:** 10.3389/fneur.2026.1815899

**Published:** 2026-04-23

**Authors:** María Zurdo-López, Miriam Sagredo del Rio, Matilde Cháfer Rudilla, Antonio Ibarra, Ernesto Doncel-Pérez

**Affiliations:** 1 Laboratorio de Bioquímica Clínica, Hospital General Universitario de Toledo, Servicio de Salud de Castilla-La Mancha (SESCAM), Toledo, Spain; 2 Centro de Investigación en Ciencias de la Salud (CICSA), Facultad de Ciencias de la Salud, Campus Norte de la Universidad Anáhuac, Huixquilucan, Estado de México, Mexico; 3 Grupo de Regeneración Neural, Hospital Nacional de Parapléjicos, Servicio de Salud de Castilla-La Mancha (SESCAM), Instituto de Investigación Sanitaria de Castilla-La Mancha (IDISCAM), Toledo, Spain

**Keywords:** biomarkers, Guillain–Barré syndrome, gut microbiota, microbial metabolites, neuroprotection, short-chain fatty acids

## Abstract

Guillain-Barré syndrome (GBS) is an acute autoimmune polyradiculoneuropathy that follows infection and is characterized by immune-mediated demyelination or axonal injury of the peripheral nervous system. While established triggers such as *Campylobacter jejuni* are well recognized, increasing evidence implicates the gut microbiota as a key modulator of immune responses relevant to GBS pathogenesis. The intestinal microbiota produces a diverse array of bioactive metabolites, including short-chain fatty acids (SCFAs), tryptophan-derived indoles, and neurotransmitter-like molecules, which influence immune tolerance, gut barrier integrity, and neuroinflammatory signaling. SCFAs, particularly butyrate, exert anti-inflammatory effects and support epithelial and blood–nerve barrier function. Microbial tryptophan metabolites regulate astrocyte and microglial activity via aryl hydrocarbon receptor (AHR) signaling, thereby restraining central and peripheral neuroinflammation. In contrast, dysbiosis-associated metabolites such as lipopolysaccharide (LPS) may enhance systemic inflammation, disrupt immune tolerance, and promote autoantibody production through mechanisms including molecular mimicry. Studies suggest that specific microbial taxa and metabolite signatures may serve as diagnostic or prognostic biomarkers in GBS, offering insights into disease susceptibility and progression. Microbiota-targeted therapeutic strategies are emerging as promising adjuncts to immunotherapy. Probiotics and prebiotics may restore beneficial microbial communities and rebalance immunoregulatory metabolite production, while host-directed metabolic interventions such as creatine supplementation may further support mitochondrial function, immunometabolic homeostasis, and neuroprotection. Fecal microbiota transplantation (FMT), though still experimental in GBS, has shown benefit in related neuroinflammatory disorders by reestablishing eubiosis and dampening immune activation. Future studies integrating metagenomic, metabolomic, and immunologic profiling in well-characterized GBS cohorts are essential to validate these findings and advance personalized microbiota-based interventions.

## Introduction

Guillain–Barré syndrome (GBS) is an acute immune-mediated disorder of the peripheral nervous system and the most common cause of acute flaccid paralysis worldwide (1, 2). Clinically, GBS is characterized by rapidly progressive, symmetrical limb weakness, areflexia, and variable sensory and autonomic dysfunction (1, 3). Disease severity ranges from mild weakness to life-threatening respiratory failure requiring mechanical ventilation, with a substantial proportion of patients experiencing incomplete recovery and long-term disability.

Several clinical and electrophysiological subtypes of GBS have been described. Acute inflammatory demyelinating polyneuropathy (AIDP) predominates in Western countries, whereas axonal variants—acute motor axonal neuropathy (AMAN) and acute motor and sensory axonal neuropathy (AMSAN)—are more prevalent in Asia and Latin America and are often associated with more severe disease and poorer prognosis (1, 3).

**Figure 1:**
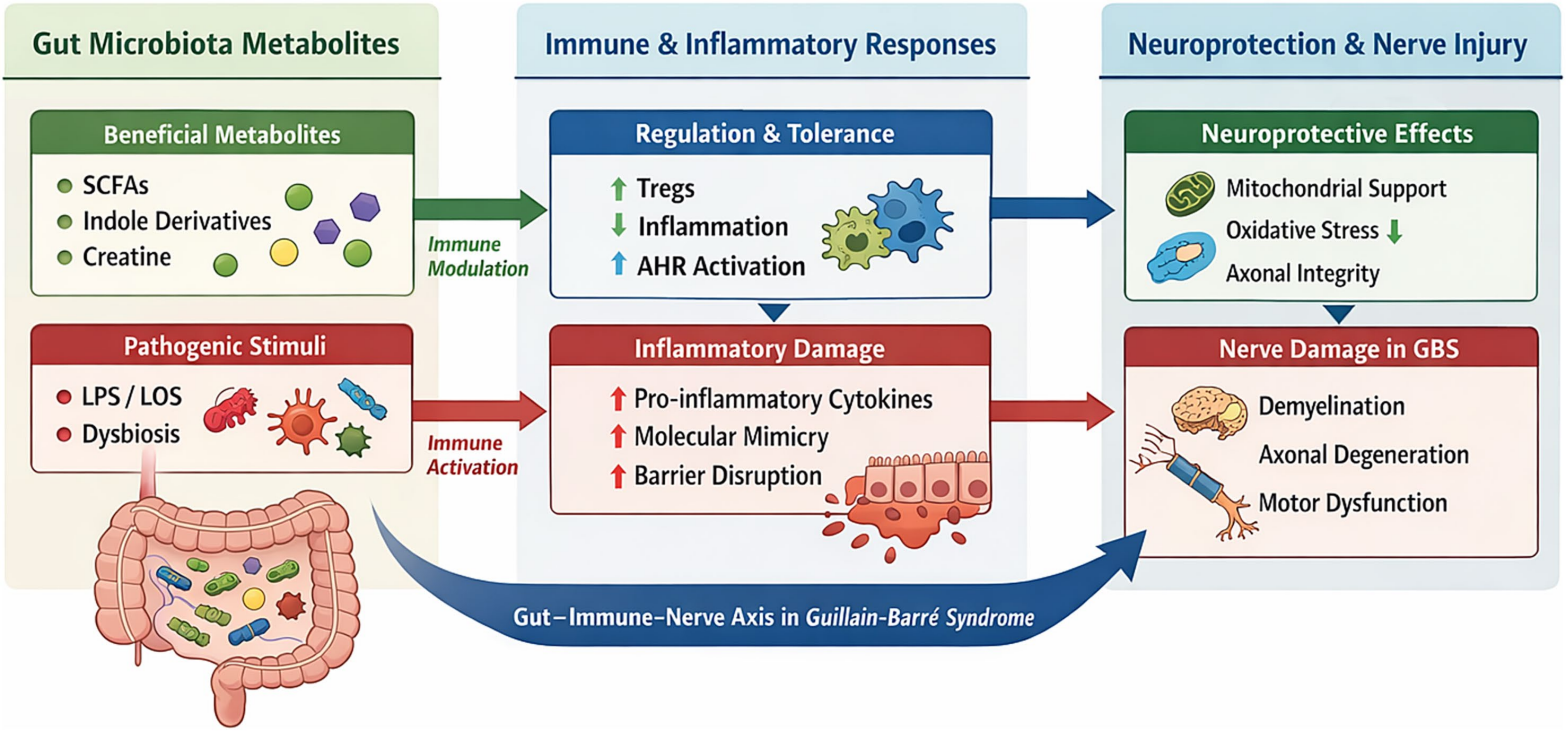
Interactions between gut microbiota-derived metabolites, immune responses, and neuroprotection in Guillain-Barre syndrome (GBS). Beneficial metabolites (SCFAs, indole derivatives, creatine) support immune regulation and neuroprotection, while pathogenic stimuli (LPS/LOS, dysbiosis) promote inflammation and nerve damage.

GBS is widely regarded as an autoimmune condition triggered by environmental factors, most notably infections (1, 4). Immune-mediated damage to peripheral nerves results from aberrant humoral and cellular responses directed against myelin or axolemmal components, leading to complement activation and inflammatory nerve injury (4–6) (Figure 1).

Molecular mimicry between microbial antigens and peripheral nerve gangliosides is considered a central pathogenic mechanism (Figure 1). Antibodies generated against microbial structures may cross-react with gangliosides such as GM1, GD1a, and GQ1b, initiating immune-mediated demyelination or axonal degeneration (4–7).

Approximately two-thirds of GBS cases are preceded by an infectious episode, most commonly involving the gastrointestinal or respiratory tract (8, 9). *Campylobacter jejuni* infection is the strongest and most consistently associated trigger, particularly for axonal subtypes (9, 10). Other implicated pathogens include cytomegalovirus, Epstein–Barr virus, influenza virus, Zika virus, and *Mycoplasma pneumoniae* (7, 9, 11). Rare associations with vaccination or surgery have been reported, although causal links remain controversial (12).

The gut microbiota plays a fundamental role in immune maturation, tolerance, and systemic immune regulation (13–15). Dysbiosis has been implicated in multiple autoimmune and neuroinflammatory diseases (16–18) Given the immune-mediated nature of GBS and its strong association with infections, the microbiota has emerged as a potential modifier of disease susceptibility, severity, and recovery (18, 19) (Figure 1).

### Geographical variability, diet, and microbiota in GBS

Guillain-Barré syndrome exhibits notable geographical variability in its clinical subtypes, with acute inflammatory demyelinating polyneuropathy (AIDP) predominating in Western countries, whereas axonal variants such as acute motor axonal neuropathy (AMAN) and acute motor and sensory axonal neuropathy (AMSAN) are more frequently observed in Asia and Latin America. These differences have been traditionally attributed to variations in infectious triggers, particularly *Campylobacter jejuni*, but may also reflect underlying differences in host–microbiota interactions.

Dietary patterns, a major determinant of gut microbiota composition, vary substantially across regions and may influence immune homeostasis. Western dietary patterns, typically high in fat and refined sugars and low in fiber, have been associated with reduced microbial diversity, decreased abundance of short-chain fatty acid (SCFA)-producing bacteria such as *Faecalibacterium* and *Roseburia*, and increased representation of pro-inflammatory taxa. In contrast, traditional or rural diets rich in fiber are linked to greater microbial diversity and enrichment of fermentative genera such as *Prevotella*, which contribute to SCFA production and immune regulation (20, 21). Although these studies are not specific to GBS, they support the concept that diet-driven microbiota differences may shape immune responses and potentially influence disease susceptibility and clinical phenotypes.

Emerging evidence also suggests a potential link between gut microbiota composition and GBS susceptibility. While direct microbiome studies in GBS patients remain limited and heterogeneous, genetic approaches such as Mendelian randomization analyses (which use genetic variants as instrumental variables to infer potential causal relationships) have identified associations between specific bacterial taxa and disease risk. For instance, genera including *Ruminococcus*, *Eubacterium*, and *Romboutsia* have been associated with increased susceptibility to GBS, whereas taxa within the *Lachnospiraceae* family may exert protective effects (22, 23). Importantly, these findings do not establish direct causality at the functional level; but they provide further support for a role of gut microbiota in modulating host immune responses relevant to GBS pathogenesis.

Together, these observations suggest that geographical, dietary, and microbiota-related factors may contribute to the heterogeneity of GBS phenotypes and immune responses. Integrating environmental and host-specific determinants, including diet and microbiota composition, may therefore be essential to better understand disease variability and to identify novel therapeutic or preventive strategies.

### Microbiota alterations in Guillain–Barré syndrome

Direct studies of the gut microbiota in GBS remain limited, but available evidence suggests reduced microbial diversity and shifts in specific bacterial taxa compared with healthy controls (24–26). These alterations resemble dysbiotic patterns observed in other autoimmune disorders.

Notably, reduced abundance of short-chain fatty acid (SCFA)-producing bacteria such as *Faecalibacterium prausnitzii* or *Roseburia hominis*, alongside increases in pro-inflammatory taxa, has been reported in dysbiotic gut microbiota profiles of inflammatory diseases compared with healthy controls (27–29). Such changes may promote systemic inflammation and lower the threshold for autoimmune activation. Whether these alterations precede disease onset or reflect consequences of acute illness or treatment remains unresolved.

### Microbial metabolites and neuroimmune regulation

#### Short-chain fatty acids

Gut bacteria ferment dietary fibers into short-fatty acids (SCFAs), principally acetate, propionate, and butyrate. These SCFAs modulate immune regulation, enhance gut barrier integrity, and influence blood-nerve barrier function. In neurodegenerative diseases SCFAs help reduce microglial activation, oxidative stress, preserve neuronal health, and counter neuroinflammation (30). Though direct studies in GBS are scarce, SCFAs likely dampen systemic and neural inflammatory cascades that are central to GBS pathogenesis (Table 1).

**Table 1:** Gut microbiota– and host metabolism–derived factors influencing immune regulation and neuroprotection in Guillain–Barré syndrome.

Source	Metabolite/component	Main immunological or neurobiological effects	Relevance to GBS and neuroprotection
SCFA-producing bacteria (*Faecalibacterium prausnitzii, Roseburia spp*)	Butyrate, propionate, acetate	Induction of regulatory T cells; suppression of pro-inflammatory cytokines; reinforcement of epithelial and blood–nerve barrier integrity; modulation of microglial activation	Reduced immune tolerance and prolonged neuroinflammation when depleted; potential contribution to secondary nerve damage (27–31)
Tryptophan-metabolizing bacteria	Indole derivatives (e.g., indole-3-propionic acid)	AHR-mediated regulation of innate and adaptive immunity; restraint of neuroinflammatory signaling; support of immune homeostasis	Dysregulated AHR signaling may favor exaggerated immune responses and peripheral nerve injury (33–35)
Gram-negative bacteria (including *Campylobacter jejuni*)	LPS/LOS	Innate immune activation via TLR4; promotion of systemic inflammation; molecular mimicry through ganglioside-like LOS structures	Synergistic amplification of molecular mimicry and inflammatory damage in susceptible individuals (17, 36–39)
Host-derived metabolic substrate	Creatine	ATP buffering; mitochondrial stabilization; reduction of oxidative stress; immunometabolic modulation of macrophage and T-cell responses	Support of axonal resilience, neuromuscular recovery, and neuroprotection as an adjunctive strategy (40, 47–50)

SCFAs, exert potent immunomodulatory effects by promoting regulatory T cell differentiation, suppressing pro-inflammatory cytokine production, and enhancing BBB integrity (15, 27, 30, 31). Reduced SCFA production in GBS-associated dysbiosis may impair immune tolerance and prolong inflammation (Table 1).

#### Tryptophan metabolites and AHR signaling

Gut bacteria metabolize dietary tryptophan into indole and related metabolites (e.g., indole-3-propionic acid, indole-3-aldehyde) that act as agonists of the aryl hydrocarbon receptor (AHR). These AHR ligands are expressed on astrocytes and microglia and have been shown to restrain central nervous system inflammation by modulating inflammatory gene programs in glial cells (32–34). Administration of indole, has been shown to promote adult hippocampal neurogenesis in mice via the AHR pathway, and indole-related metabolites activate AHR signaling to mitigate neuroinflammatory responses in CNS disease models (35). These effects in suppressing neuroinflammatory signaling may be relevant to autoimmune nerve injury in GBS. Subsequently, microbiota-derived tryptophan metabolites activate the AHR, a key regulator of immune homeostasis and neuroinflammation (32–34). Dysregulation of this pathway may contribute to exaggerated immune responses and peripheral nerve injury in GBS (Table 1).

#### Lipopolysaccharide and pro-inflammatory signals

Increased exposure to lipopolysaccharide (LPS) due to dysbiosis or impaired gut barrier function can activate innate immune responses and enhance autoimmunity (17, 32). In GBS, LPS-driven inflammation may synergize with molecular mimicry mechanisms following infection. Lipopolysaccharides, more precisely lipooligosaccharides (LOS), from *Campylobacter jejuni* and other Gram-negative pathogens share structural homology with peripheral nerve gangliosides, providing a well-established basis for molecular mimicry in Guillain-Barré syndrome. Early studies demonstrated that LOS from *C. jejuni* isolated from GBS patients contain ganglioside-like epitopes that induce cross-reactive anti-ganglioside antibodies, leading to complement activation and immune-mediated nerve injury (36, 37). Beyond antigenic mimicry, LPS acts as a potent pro-inflammatory trigger through Toll-like receptor 4 (TLR4) signaling, amplifying innate immune activation and cytokine release, which may lower the threshold for autoreactive B- and T-cell responses (38). This inflammatory milieu likely enhances antibody production and effector mechanisms, thereby potentiating nerve damage in susceptible individuals (39). Together, these findings support a synergistic model in which LPS-driven inflammation and molecular mimicry cooperate to initiate and propagate immune-mediated peripheral neuropathy in GBS (Table 1).

### Microbial and metabolomic biomarkers in GBS

Microbiota composition and metabolite profiles represent promising biomarkers for disease susceptibility, severity, and prognosis. Altered SCFA levels, disrupted tryptophan metabolism, and markers of endotoxemia may correlate with immune activity and clinical outcomes (24–26). Longitudinal, multi-omics studies are needed to validate these candidates.

### Emerging microbiota-targeted therapeutic strategies

#### Probiotics and prebiotics

Microbiota-targeted interventions such as probiotics and prebiotics have gained increasing attention as potential adjunctive strategies in immune-mediated neurological disorders. Probiotics may promote the expansion of beneficial commensal bacteria, enhance short-chain fatty acid (SCFA) production, and restore immune tolerance by modulating regulatory T cell responses and cytokine profiles (15, 18). Prebiotics, by providing fermentable substrates for commensal bacteria, may further support these effects by selectively enriching SCFA-producing taxa (15, 27).

Although controlled clinical trials evaluating probiotics or prebiotics in Guillain–Barré syndrome are currently lacking, evidence from other autoimmune and neuroinflammatory conditions suggests that these interventions may attenuate systemic inflammation and improve immune homeostasis (18, 19, 27). Given the relative safety and accessibility of these approaches, they represent promising candidates for future investigation as supportive therapies during recovery or remission phases of GBS.

#### Postbiotics and microbial metabolite supplementation

Postbiotics—defined as non-viable microbial products or metabolites with biological activity—offer a targeted approach to modulate host immune responses without administering live microorganisms. Among these, SCFAs such as butyrate have demonstrated potent anti-inflammatory and barrier-supporting properties, including promotion of regulatory T cell differentiation and suppression of pro-inflammatory signaling pathways (15, 27).

In the context of GBS, supplementation with SCFAs or SCFA-mimicking compounds could theoretically compensate for dysbiosis-associated deficits in immunoregulatory metabolites observed in some patients (24–26). While direct evidence in GBS is currently unavailable, preclinical and clinical data from other autoimmune neurological disorders support the concept that metabolite-based interventions may contribute to immune rebalancing and neuroprotection (18, 27).

#### Fecal microbiota transplantation

Fecal microbiota transplantation (FMT) represents the most comprehensive strategy to restore microbial ecosystem balance by transferring a complete microbial community from a healthy donor to a recipient. FMT has demonstrated efficacy in recurrent Clostridioides difficile infection and has shown promising immunomodulatory effects in selected autoimmune and neuroinflammatory disorder (18, 19).

To date, FMT has not been systematically evaluated in GBS. The acute onset, rapid progression, and frequent need for intensive care pose significant challenges regarding safety, timing, and patient selection. Nevertheless, in carefully controlled settings and possibly during the recovery phase, FMT may offer a future avenue to re-establish eubiosis and dampen persistent immune activation. Rigorous clinical studies are required before this approach can be considered in GBS.

#### Dietary interventions

Dietary modulation represents a non-invasive and widely applicable strategy to influence gut microbiota composition and metabolic output. Diets rich in fermentable fibers, polyphenols, and anti-inflammatory nutrients have been shown to enhance SCFA production and support immune tolerance (15, 27).

In GBS, dietary interventions may serve as supportive measures to promote microbiota resilience during recovery, particularly following antibiotic exposure or prolonged hospitalization. While dietary strategies alone are unlikely to replace immunotherapy, they may complement existing treatments by reinforcing microbiota-mediated immune regulation.

#### Creatine supplementation as a metabolically mediated neuroprotective strategy

Beyond microbiota-derived metabolites, host-derived metabolic substrates may also influence immune and neuroprotective responses in Guillain–Barré syndrome (GBS). Creatine is a key component of cellular energy metabolism, acting as a phosphate buffer that maintains adenosine triphosphate (ATP) availability in tissues with high energetic demand, including peripheral nerves and skeletal muscle (40). Experimental studies have demonstrated that creatine exerts neuroprotective effects through mitochondrial stabilization, reduction of oxidative stress, and preservation of axonal integrity under conditions of metabolic stress (41, 42) (Table 1).

In addition to its bioenergetic role, creatine has emerged as an immunometabolic modulator, with creatine availability shown to influence macrophage polarization and cytokine production, as well as enhance macrophage-mediated support of effector T-cell responses, suggesting a role in shaping immune responses relevant to autoimmune and inflammatory disorders (43, 44). Notably, creatine uptake via the creatine transporter (SLC6A8) supports cellular resilience during immune activation by sustaining metabolic capacity in macrophages and T cells, with SLC6A8-dependent creatine transport influencing cytokine responses, effector function, and immune cell homeostasis — processes that may limit excessive inflammatory damage under conditions of sustained activation (43, 45, 46).

Although direct clinical evidence for creatine supplementation in GBS is currently lacking, beneficial effects have been reported in neuromuscular and neurodegenerative conditions characterized by axonal dysfunction, mitochondrial impairment, or muscle wasting (40, 47, 48). These properties suggest that creatine supplementation could represent a safe and accessible adjunctive strategy to support nerve recovery, neuromuscular function, and rehabilitation in GBS, particularly in axonal subtypes (Table 1).

Importantly, creatine metabolism may also interact indirectly with gut microbial ecology and intestinal barrier function through shared amino acid pathways involving arginine and glycine metabolism, further supporting its inclusion within a broader host–microbiota–metabolism framework (49, 50). Well-designed clinical studies are required to evaluate the optimal timing, dosage, and efficacy of creatine supplementation in GBS patients.

### Immunomodulatory therapeutic strategies beyond microbiota

Guillain-Barré syndrome is primarily an antibody-mediated disease, in which anti-ganglioside antibodies play a central role in nerve damage through complement activation and interaction with immune effector cells. Accordingly, current therapeutic strategies are largely aimed at interfering with the production and pathogenic activity of these antibodies. First-line treatments such as intravenous immunoglobulin (IVIg) and plasmapheresis act by neutralizing circulating autoantibodies and modulating the immune response. In addition, emerging approaches targeting humoral immunity include complement inhibition and blockade of the neonatal Fc receptor (FcRn), which regulates IgG recycling and persistence (51, 52).

#### Distinct therapeutic mechanisms of IVIg and plasmapheresis

Despite their shared clinical efficacy, IVIg and plasmapheresis rely on fundamentally different mechanisms of action. Plasmapheresis primarily exerts its effect through the physical removal of circulating pathogenic factors, including autoantibodies, complement components, and inflammatory mediators. In contrast, IVIg exerts broader immunomodulatory effects beyond the neutralization of autoantibodies. One of its key mechanisms involves anti-idiotypic antibodies capable of binding and functionally blocking pathogenic anti-ganglioside antibodies at the peripheral nerve level. IVIg has also been shown to interfere with complement activation, thereby limiting membrane attack complex formation and subsequent axonal injury. Furthermore, competition for Fc receptor binding on immune effector cells promotes inhibitory signaling pathways, reducing inflammatory activation and downstream tissue damage (53).

#### IVIg therapy in gut microbiota and immune-mediated diseases

In this context, the role of the gut microbiota in immune-mediated neuropathies remains complex and, in some aspects, controversial. In patients with chronic inflammatory demyelinating polyneuropathy (CIDP) receiving IVIg, the gut microbiome exhibits distinct features compared to healthy individuals, including increased bacterial diversity and enrichment of Firmicutes such as *Blautia*, *Eubacterium hallii*, and *Ruminococcus torques*, as well as Actinobacteriota, with a predominance of short-chain fatty acid-producing taxa. Notably, IVIg does not appear to induce significant short-term changes in gut microbiome composition, suggesting that its therapeutic effects are not mediated through rapid microbiota modulation (54).

Insights from other immune-mediated diseases further support the interplay between gut barrier integrity and systemic immune responses. In Kawasaki disease (KD), increased intestinal permeability and disruption of gut immune homeostasis have been associated with endothelial dysfunction, platelet activation, and the development of vascular complications. Importantly, gastrointestinal involvement has been linked to reduced responsiveness to IVIg therapy, suggesting that gut-derived immune alterations may influence treatment outcomes (55). Taken together, these observations support a model in which, although IVIg primarily exerts its effects through immunomodulation of pathogenic antibodies and complement pathways, host–microbiota interactions and gut barrier status may modulate disease severity and therapeutic responsiveness in immune-mediated disorders, including Guillain-Barré syndrome. These findings highlight the need for integrated therapeutic approaches targeting both humoral immunity and host–microbiota interactions in GBS.

In addition to established therapies, alternative strategies aimed at directly interfering with pathogenic autoantibodies have been proposed. One conceptual approach involves the use of ganglioside-mimicking or lipooligosaccharide (LOS)-derived epitopes to competitively inhibit the binding of anti-ganglioside antibodies to peripheral nerves, based on the molecular mimicry mechanisms underlying GBS (6, 7). Although still largely experimental, this strategy may offer a targeted means to neutralize pathogenic antibodies. Furthermore, the use of anti-idiotypic antibodies represents another potential therapeutic avenue, as these antibodies can bind to the antigen-binding sites of pathogenic autoantibodies and prevent their interaction with neural targets. This mechanism is thought to contribute, at least in part, to the therapeutic effects of intravenous immunoglobulin (5, 53). These approaches highlight the potential for more specific immunotherapies aimed at directly blocking autoimmune effector mechanisms in GBS.

### Future directions and neuroprotective perspectives

Despite significant advances in the understanding of Guillain–Barré syndrome pathogenesis, current therapeutic approaches remain largely reactive and focused on acute immunomodulation through intravenous immunoglobulin or plasma exchange (1, 9). These therapies effectively reduce disease severity but do not directly address upstream factors that influence immune susceptibility, disease heterogeneity, or long-term recovery.

Emerging evidence suggests that the gut microbiota and its metabolic output represent key modulators of systemic and neuroimmune responses relevant to peripheral nerve injury (15, 18). Integrating microbiota-informed perspectives into GBS research may enable a shift toward earlier risk stratification, improved prognostic assessment, and the development of personalized therapeutic strategies.

Future studies should prioritize longitudinal, multi-omics approaches combining microbiome profiling, metabolomics, and immune phenotyping in well-characterized GBS cohorts (24–26, 48). Such integrative analyses may help determine whether microbiota alterations precede disease onset, modulate disease severity, or influence recovery trajectories. Identification of reproducible microbial or metabolomic signatures could facilitate the development of biomarkers for disease monitoring and therapeutic response.

From a neuroprotective standpoint, microbiota-derived metabolites such as SCFAs and tryptophan-derived indoles represent particularly attractive targets due to their capacity to promote immune tolerance, reinforce barrier integrity, and restrain excessive inflammation through pathways such as AHR signaling (15, 32–34). Modulating these pathways may help limit secondary nerve damage and support regenerative processes following acute immune-mediated injury.

## Conclusion

From a neuroprotective and metabolic perspective, host-directed strategies deserve increasing attention in Guillain–Barré syndrome (GBS). Creatine supplementation, by supporting mitochondrial function, buffering cellular energy failure, and modulating immunometabolic pathways, represents a promising adjunctive approach to complement conventional immunotherapies. Future research should determine whether creatine can enhance axonal resilience, improve functional recovery, and synergize with rehabilitation programs or microbiota-targeted interventions. More broadly, integrating metabolic substrates with microbiome-based modulation within multimodal therapeutic frameworks may provide a more comprehensive strategy for GBS management, simultaneously addressing immune dysregulation, host–microbe interactions, and neuronal energy vulnerability. Although these concepts remain largely exploratory, they open new avenues for neuroprotective interventions aligned with precision medicine, systems immunology, and personalized neurorehabilitation in immune-mediated peripheral neuropathies.

## References

[1] Willison HJ Jacobs BC van Doorn PA. Guillain-Barré syndrome. Lancet. (2016) 388:717–27. doi: 10.1016/S0140-6736(16)00339-1, 26948435

[2] Hernández-Jardón CH Velásquez-Pérez L. Epidemiological and clinical aspects of Guilain-Barré syndrome, 2012-2022. Rev Med Inst Mex Seguro Soc. (2024) 62:1–7. doi: 10.5281/zenodo.13306747, 39570324 PMC12520721

[3] Akbayram S Doğan M Akgün C Peker E Sayιn R Aktar F . Clinical features and prognosis with Guillain-Barré syndrome. Ann Indian Acad Neurol. (2011) 14:98–102. doi: 10.4103/0972-2327.82793, 21808470 PMC3141496

[4] Yuki N. Molecular mimicry and Guillain-Barré syndrome. Brain Nerve. (2015) 67:1341–6. doi: 10.11477/mf.1416200304 26560949

[5] Goodfellow JA Willison HJ. Gangliosides and autoimmune peripheral nerve diseases. Prog Mol Biol Transl Sci. (2018) 156:355–82. doi: 10.1016/bs.pmbts.2017.12.010, 29747820

[6] Laman JD Huizinga R Boons G-J Jacobs BC. Guillain-Barré syndrome: expanding the concept of molecular mimicry. Trends Immunol. (2022) 43:296–308. doi: 10.1016/j.it.2022.02.003, 35256276 PMC9016725

[7] Yuki N. Infectious origins of, and molecular mimicry in, Guillain-Barré and fisher syndromes. Lancet Infect Dis. (2001) 1:29–37. doi: 10.1016/S1473-3099(01)00019-6, 11871407

[8] Jacobs BC Rothbarth PH van der Meché FG Herbrink P Schmitz PI de Klerk MA . The spectrum of antecedent infections in Guillain-Barré syndrome: a case-control study. Neurology. (1998) 51:1110–5. doi: 10.1212/wnl.51.4.1110 9781538

[9] Leonhard SE van der Eijk AA Andersen H Antonini G Arends S Attarian S . An international perspective on preceding infections in Guillain-Barré syndrome: the IGOS-1000 cohort. Neurology. (2022) 99:e1299–313. doi: 10.1212/WNL.0000000000200885, 35981895

[10] Ramesh A Subbarayan R Srinivasan D Balakrishnan R Shrestha R Chauhan A. Infectious triggers and immune dynamics in Guillain-Barré syndrome: revisiting campylobacter jejuni and the silent role of *Haemophilus influenzae*. Microbiology. (2025) 14:e70177. doi: 10.1002/mbo3.70177, 41310913 PMC12660056

[11] Cao-Lormeau V-M Blake A Mons S Lastère S Roche C Vanhomwegen J . Guillain-Barré syndrome outbreak associated with Zika virus infection in French Polynesia: a case-control study. Lancet (London, England). (2016) 387:1531–9. doi: 10.1016/S0140-6736(16)00562-6, 26948433 PMC5444521

[12] Wachira VK Peixoto HM de Oliveira MRF. Systematic review of factors associated with the development of Guillain-Barré syndrome 2007-2017: what has changed? Trop Med Int Health. (2019) 24:132–42. doi: 10.1111/tmi.13181, 30444562

[13] Silva YP Bernardi A Frozza RL. The role of short-chain fatty acids from gut microbiota in gut-brain communication. Front Endocrinol (Lausanne). (2020) 11:25. doi: 10.3389/fendo.2020.00025, 32082260 PMC7005631

[14] Dalile B Van Oudenhove L Vervliet B Verbeke K. The role of short-chain fatty acids in microbiota-gut-brain communication. Nat Rev Gastroenterol Hepatol. (2019) 16:461–78. doi: 10.1038/s41575-019-0157-3, 31123355

[15] Cortés M Olate P Rodriguez R Diaz R Martínez A Hernández G . Human microbiome as an Immunoregulatory Axis: mechanisms, Dysbiosis, and therapeutic modulation. Microorganisms. (2025) 13:34571. doi: 10.3390/microorganisms13092147, 41011478 PMC12472381

[16] Mousa WK Chehadeh F Husband S. Microbial dysbiosis in the gut drives systemic autoimmune diseases. Front Immunol. (2022) 13:906258. doi: 10.3389/fimmu.2022.906258, 36341463 PMC9632986

[17] Stolzer I Scherer E Süß P Rothhammer V Winner B Neurath MF . Impact of microbiome-brain communication on Neuroinflammation and neurodegeneration. Int J Mol Sci. (2023) 24:14925. doi: 10.3390/ijms241914925, 37834373 PMC10573483

[18] Li J Wan B Zhou L Qian X Wang F Gu S . Gut microbiota dysbiosis induces neuroinflammation in major depressive disorders: mechanisms targeting the gut-brain axis. Front Psych. (2025) 16:1629182. doi: 10.3389/fpsyt.2025.1629182, 41048915 PMC12490329

[19] Chakrawarti A Cromarty RT Basting CM Anderson J Schroeder TA Escandon K . Pre-treatment microbiome diversity and function is associated with expansion of cytotoxic and regulatory immune populations after N-803 treatment in people with HIV. bioRxiv Prepr Serv Biol. (2025). doi: 10.1101/2025.10.01.679827

[20] Soldán M Argalášová Ľ Hadvinová L Galileo B Babjaková J. The effect of dietary types on gut microbiota composition and development of non-communicable diseases: a narrative review. Nutrients. (2024) 16:3134. doi: 10.3390/nu16183134, 39339734 PMC11434870

[21] Daunizeau C Franck M Boutin A Ruel M Poliakova N Ayotte P . The gut microbiota of indigenous populations in the context of dietary westernization: a systematic review and meta-analysis. Front Nutr. (2025) 12:1652598. doi: 10.3389/fnut.2025.1652598, 41245412 PMC12612841

[22] Cao F Zhang H Xu B Li C. Genetic association between gut microbiota and the risk of Guillain-Barré syndrome. J Affect Disord. (2024) 357:171–8. doi: 10.1016/j.jad.2024.05.011, 38703912

[23] Zhang M Fang J Zheng C Lin Q Zhang J. Gut microbiota and autoimmune neurologic disorders: a two-sample bidirectional Mendelian randomization study. Front Microbiol. (2024) 15:1337632.. doi: 10.3389/fmicb.2024.1337632, 38721606 PMC11076763

[24] Lee S Raza S Lee E-J Chang Y Ryu S Kim H-L . Metagenome-assembled genomes reveal microbial signatures and metabolic pathways linked to coronary artery disease. mSystems. (2025) 10:e0095425. doi: 10.1128/msystems.00954-25, 41196050 PMC12710354

[25] Crosby CM Kronenberg M. Tissue-specific functions of invariant natural killer T cells. Nat Rev Immunol. (2018) 18:559–74. doi: 10.1038/s41577-018-0034-2, 29967365 PMC6343475

[26] Nian D Shi P Qu H Sun J Li Q Li Q . Pathogenic mechanism of intestinal microbiota involved in Guillain-Barré syndrome and with *Bifidobacterium* intervention. Arch Med Sci. (2025) 21:1051–61. doi: 10.5114/aoms/128103, 40741279 PMC12305515

[27] Cao Y Shen J Ran ZH. Association between *Faecalibacterium prausnitzii* reduction and inflammatory bowel disease: a Meta-analysis and systematic review of the literature. Gastroenterol Res Pract. (2014) 2014:872725. doi: 10.1155/2014/872725, 24799893 PMC3985188

[28] Fujimoto T Imaeda H Takahashi K Kasumi E Bamba S Fujiyama Y . Decreased abundance of *Faecalibacterium prausnitzii* in the gut microbiota of Crohn’s disease. J Gastroenterol Hepatol. (2013) 28:613–9. doi: 10.1111/jgh.12073, 23216550

[29] Machiels K Joossens M Sabino J De Preter V Arijs I Eeckhaut V . A decrease of the butyrate-producing species *Roseburia hominis* and *Faecalibacterium prausnitzii* defines dysbiosis in patients with ulcerative colitis. Gut. (2014) 63:1275–83. doi: 10.1136/gutjnl-2013-304833, 24021287

[30] Xie L Alam MJ Marques FZ Mackay CR. A major mechanism for immunomodulation: dietary fibres and acid metabolites. Semin Immunol. (2023) 66:101737. doi: 10.1016/j.smim.2023.101737, 36857894

[31] Chenghan M Wanxin L Bangcheng Z Yao H Qinxi L Ting Z . Short-chain fatty acids mediate gut microbiota-brain communication and protect the blood-brain barrier integrity. Ann N Y Acad Sci. (2025) 1545:116–31. doi: 10.1111/nyas.15299, 39998158

[32] Sittipo P Choi J Lee S Lee YK. The function of gut microbiota in immune-related neurological disorders: a review. J Neuroinflammation. (2022) 19:154. doi: 10.1186/s12974-022-02510-1, 35706008 PMC9199126

[33] Rothhammer V Mascanfroni ID Bunse L Takenaka MC Kenison JE Mayo L . Type I interferons and microbial metabolites of tryptophan modulate astrocyte activity and central nervous system inflammation via the aryl hydrocarbon receptor. Nat Med. (2016) 22:586–97. doi: 10.1038/nm.4106, 27158906 PMC4899206

[34] Rothhammer V Quintana FJ. The aryl hydrocarbon receptor: an environmental sensor integrating immune responses in health and disease. Nat Rev Immunol. (2019) 19:184–97. doi: 10.1038/s41577-019-0125-8, 30718831

[35] Wei GZ Martin KA Xing PY Agrawal R Whiley L Wood TK . Tryptophan-metabolizing gut microbes regulate adult neurogenesis via the aryl hydrocarbon receptor. Proc Natl Acad Sci USA. (2021) 118:e2021091118. doi: 10.1073/pnas.2021091118, 34210797 PMC8271728

[36] Yuki N Taki T Inagaki F Kasama T Takahashi M Saito K . A bacterium lipopolysaccharide that elicits Guillain-Barré syndrome has a GM1 ganglioside-like structure. J Exp Med. (1993) 178:1771–5. doi: 10.1084/jem.178.5.1771, 8228822 PMC2191246

[37] Yuki N. Molecular mimicry between gangliosides and lipopolysaccharides of *Campylobacter jejuni* isolated from patients with Guillain-Barré syndrome and Miller fisher syndrome. J Infect Dis. (1997) 176:S150–3. doi: 10.1086/513800 9396700

[38] Sheikh KA Ho TW Nachamkin I Li CY Cornblath DR Asbury AK . Molecular mimicry in Guillain-Barré syndrome. Ann N Y Acad Sci. (1998) 845:307–21. doi: 10.1111/j.1749-6632.1998.tb09683.x, 9668364

[39] Sheikh KA Nachamkin I Ho TW Willison HJ Veitch J Ung H . *Campylobacter jejuni* lipopolysaccharides in Guillain-Barré syndrome: molecular mimicry and host susceptibility. Neurology. (1998) 51:371–8. doi: 10.1212/wnl.51.2.371, 9710005

[40] Kreider RB. Effects of creatine supplementation on performance and training adaptations. Mol Cell Biochem. (2003) 244:89–94. doi: 10.1023/A:1022465203458 12701815

[41] Taylor MK Burns JM Choi I-Y Herda TJ Lee P Smith AN . Protocol for a single-arm, pilot trial of creatine monohydrate supplementation in patients with Alzheimer’s disease. Pilot feasibility Stud. (2024) 10:42. doi: 10.1186/s40814-024-01469-5, 38414003 PMC10898014

[42] Bender A Klopstock T. Creatine for neuroprotection in neurodegenerative disease: end of story? Amino Acids. (2016) 48:1929–40. doi: 10.1007/s00726-015-2165-0, 26748651

[43] Ji L Zhao X Zhang B Kang L Song W Zhao B . Slc6a8-mediated Creatine uptake and accumulation reprogram macrophage polarization via regulating cytokine responses. Immunity. (2019) 51:272–284.e7. doi: 10.1016/j.immuni.2019.06.007, 31399282

[44] Peng Z Saito S. Creatine supplementation enhances anti-tumor immunity by promoting adenosine triphosphate production in macrophages. Front Immunol. (2023) 14:1176956. doi: 10.3389/fimmu.2023.1176956, 37662917 PMC10471797

[45] Di Biase S Ma X Wang X Yu J Wang Y-C Smith DJ . Creatine uptake regulates CD8 T cell antitumor immunity. J Exp Med. (2019) 216:2869–82. doi: 10.1084/jem.20182044 31628186, 31628186 PMC6888972

[46] Samborska B Roy DG Rahbani JF Hussain MF Ma EH Jones RG . Creatine transport and creatine kinase activity is required for CD8+ T cell immunity. Cell Rep. (2022) 38:110446. doi: 10.1016/j.celrep.2022.110446, 35235777

[47] Takeda K Tasai M Iwamoto M Onishi A Tagami T Nirasawa K . Microinjection of cytoplasm or mitochondria derived from somatic cells affects parthenogenetic development of murine oocytes. Biol Reprod. (2005) 72:1397–404. doi: 10.1095/biolreprod.104.036129, 15716395

[48] Díaz R Blanco-García J Rodríguez-Gómez J Vargas-Baquero E Fernández-Alarcón C Terán-Tinedo JR . Hospital coordination and protocols using serum and peripheral blood cells from patients and healthy donors in a longitudinal study of Guillain-Barré syndrome. Diagnostics (Basel, Switzerland). (2025) 15:1900. doi: 10.3390/diagnostics15151900, 40804862 PMC12346330

[49] Turer E McAlpine W Wang K-W Lu T Li X Tang M . Creatine maintains intestinal homeostasis and protects against colitis. Proc Natl Acad Sci USA. (2017) 114:E1273–81. doi: 10.1073/pnas.1621400114, 28137860 PMC5321020

[50] Ostojic SM. Human gut microbiota as a source of guanidinoacetic acid. Med Hypotheses. (2020) 142:109745. doi: 10.1016/j.mehy.2020.109745, 32344286

[51] Sprenger-Svačina A Svačina MKR Gao T Zhang G Sheikh KA. Emerging treatment landscape for Guillain-Barré syndrome (GBS): what’s new? Expert Opin Investig Drugs. (2024) 33:881–6. doi: 10.1080/13543784.2024.2377323, 38980318 PMC11424254

[52] Nagelkerke SQ Kuijpers TW. Immunomodulation by IVIg and the role of fc-gamma receptors: classic mechanisms of action after all? Front Immunol. (2015) 5:674. doi: 10.3389/fimmu.2014.00674, 25653650 PMC4301001

[53] Arumugham VB Rayi A Intravenous Immunoglobulin (IVIG). Treasure Island (FL): StatPearls Publishing (2026). 32119333

[54] Svačina MKR Sprenger-Svačina A Tsakmaklis A Rüb AM Klein I Wüstenberg H . The gut microbiome in intravenous immunoglobulin-treated chronic inflammatory demyelinating polyneuropathy. Eur J Neurol. (2023) 30:3551–6. doi: 10.1111/ene.15679, 36651357

[55] Tao E Lang D. Unraveling the gut: the pivotal role of intestinal mechanisms in Kawasaki disease pathogenesis. Front Immunol. (2024) 15:1496293. doi: 10.3389/fimmu.2024.1496293, 39664384 PMC11633670

